# Quantitative evaluation of upper limb ataxia in spinocerebellar ataxias

**DOI:** 10.1002/acn3.51528

**Published:** 2022-03-15

**Authors:** Yoshiyuki Kishimoto, Atsushi Hashizume, Yuta Imai, Masahiro Nakatochi, Shinichiro Yamada, Daisuke Ito, Ryota Torii, Yoshitaka Nagano, Hideo Fujimoto, Masahisa Katsuno

**Affiliations:** ^1^ Department of Neurology Nagoya University Graduate School of Medicine Nagoya Japan; ^2^ Department of Clinical Research Education Nagoya University Graduate School of Medicine Nagoya Japan; ^3^ Department of Basic Medicinal Sciences Nagoya University Graduate School of Pharmaceutical Sciences Nagoya Japan; ^4^ Public Health Informatics Unit, Department of Integrated Health Sciences Nagoya University Graduate School of Medicine Nagoya Japan; ^5^ Department of Electronic Robot Engineering Aichi University of Technology Gamagori Japan; ^6^ Department of Computer Science and Engineering Nagoya Institute of Technology Graduate School of Engineering Nagoya Japan

## Abstract

**Objective:**

To quantitatively evaluate upper limb ataxia using a novel pen‐like sensor device in patients with spinocerebellar ataxia (SCA) and to assess its validity, reliability, and sensitivity to disease progression.

**Methods:**

We designed a cross‐sectional and longitudinal study of patients with SCA and healthy controls. Upper limb ataxia was evaluated using a device that measures the three‐dimensional position every 10 msec. Participants were instructed to move a pen‐like part of the device iteratively between two buttons. We evaluated the time, length, velocity, and variation coefficient of the stroke, and calculated the distortion index using the mean squared error. The following scales were also evaluated: Scale for the Assessment and Rating of Ataxia (SARA), the International Cooperative Ataxia Rating Scale (ICARS), and the nine‐hole pegboard test. Subjects were followed 12 months after the baseline evaluation.

**Results:**

A total of 42 patients with SCA and 33 healthy controls were enrolled and evaluated. For all ataxia indices measured using the device there were significant differences between healthy controls and patients with SCA. Among the ataxia indices, the distortion index showed the strongest correlation with the SARA and ICARS upper limb score (Pearson's *r* = 0.647 and 0.722, respectively). Test–retest reliability was high for most of the ataxia indices. In the longitudinal analysis, the distortion index showed high standardized response mean and adjusted effect size, regardless of disease severity.

**Interpretation:**

Our study demonstrated that the distortion index is a reliable functional marker that is sensitive to longitudinal change in patients with SCA.

## Introduction

Spinocerebellar ataxia (SCA) is a subset of hereditary progressive neurodegenerative disorders with ataxia, characterized by unsteadiness of gait, abnormal eye movements, slurred speech, and impaired coordination of limb movements.^1^ In addition to the symptom of ataxia, SCA can also present with various non‐ataxia symptoms, such as pyramidal signs, extrapyramidal signs, sensory disturbances, and brainstem oculomotor signs.^2^ The onset of SCA is usually in adult life and the progression is usually slow.^3^

There is currently no effective treatment to slow the progression of SCA, although several clinical trials for potential disease‐modifying treatments, including cell therapy, are now underway.^2^, ^4^, ^5^, ^6^ The quantification of disease severity is essential to conduct clinical research. For SCA, the scale for the assessment and rating of ataxia (SARA) and the International Cooperative Ataxia Rating Scale (ICARS) have been used widely to quantitatively analyze motor deficits.^7^, ^8^ These rating scales are advantageous in that they are capable of evaluating total physical function and require no specific instruments for evaluation. However, their sensitivity to disease progression is limited.^3^, ^9^, ^10^ Moreover, previous reports have indicated that rating scales related to upper limb function, such as the upper limb kinetic subscales of the ICARS, are less favorable because of inadequate inter‐rater reliability.^8^, ^11^ This is probably because of difficulties in visually evaluating how patients' upper limb movements deviate from the ideal trajectory. Several quantitative methods to evaluate ataxia have been developed^12^, ^13^, ^14^, ^15^, ^16^, ^17^; however, few studies have established an evaluation method of upper limb ataxia that is sensitive to disease progression.

The present cross‐sectional and longitudinal study aimed to develop a quantitative evaluation method for upper limb ataxia for patients with SCA using a novel pen‐like sensor device and to evaluate the validity, reliability, and sensitivity to disease progression of the measured indices.

## Methods

### Standard protocol approvals, registrations, and patient consent

This study was conducted according to the Declaration of Helsinki, the Ethics Guidelines for Human Genome/Gene Analysis Research, and the Ethical Guidelines for Medical and Health Research Involving Human Subjects endorsed by the Japanese government. The study protocol was approved by the ethics review committee of Nagoya University Graduate School of Medicine. All participants were informed of the purpose of the study and provided written informed consent.

### Participants

Patients who were clinically or genetically diagnosed with SCA were recruited. The principal inclusion criteria were as follows: (1) genetically confirmed SCA or (2) cerebellar ataxia, which was inherited as an autosomal dominant trait. Patients with secondary cerebellar ataxia caused by alcohol, drugs, infarction, autoimmune diseases, or paraneoplastic syndromes were excluded. Patients who had severe complications, including heart, or respiratory failure or cognitive dysfunction were excluded. Age‐and sex‐matched healthy controls (HCs) with no history of neurological abnormalities were also recruited. All participants were evaluated at Nagoya University Hospital from November 2017 to April 2020.

### Instrument and evaluation procedure

**Figure 1:**
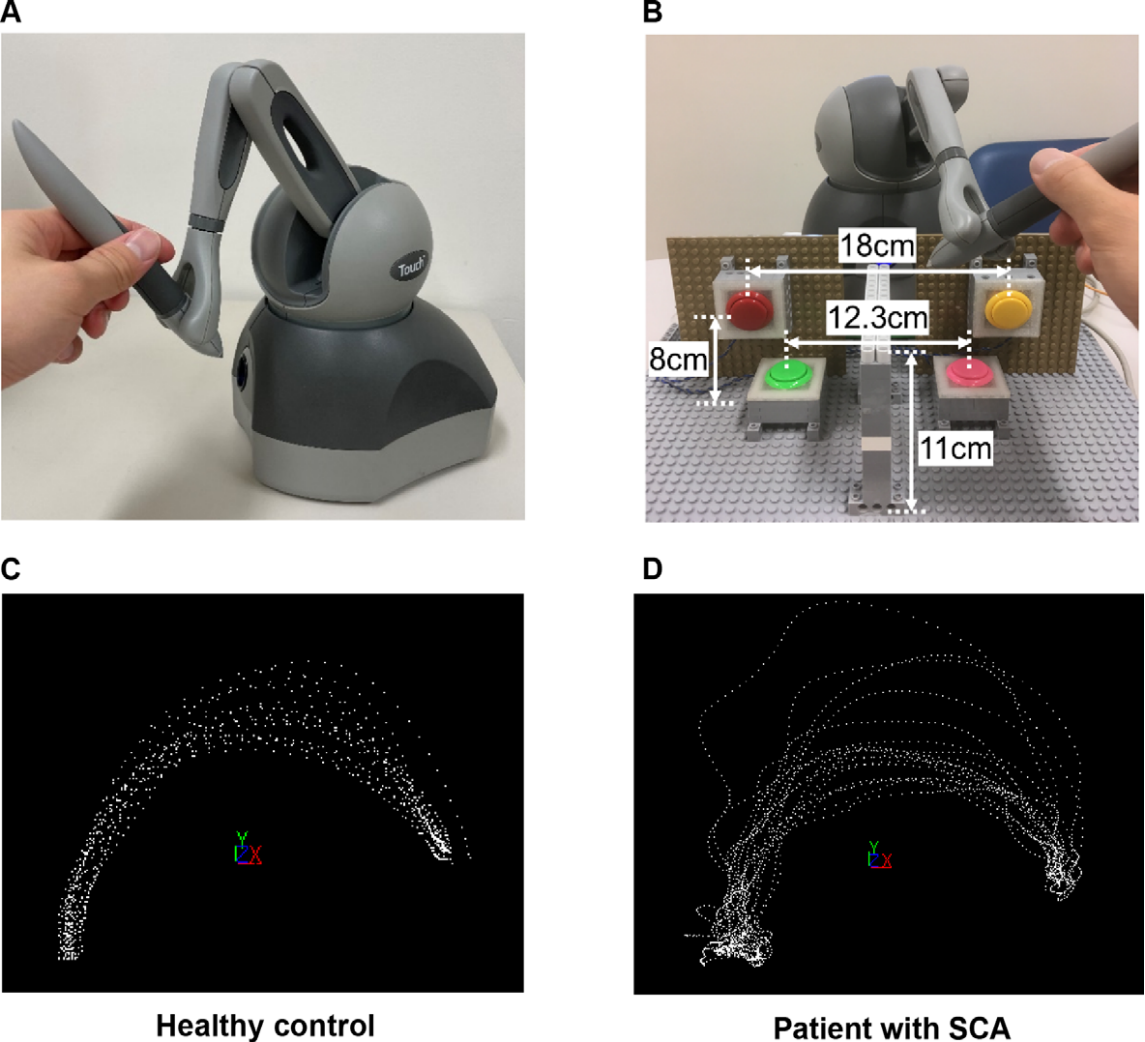
Experimental setup of the device and plot of the trajectories. Geomagic Touch (A), four buttons, and one wall were arranged for experiment (B). Dots represented the position of the top of pen‐like part every 10 msec when a healthy control (C) and a patient with SCA (D) moved it to and fro between yellow and green buttons. SCA, spinocerebellar ataxia. [Colour figure can be viewed at wileyonlinelibrary.com]

We developed a measurement system that consisted of a commercially available device (Geomagic Touch, 3D Systems, Inc., SC, USA), which accurately measures a three‐dimensional position and applies force feedback on the uses' hand, and four buttons (Fig. 1A and B). This time the function of force feedback was not used and the measurement function was used. The three‐dimensional position of the pen‐like part of the device was accurately recorded every 10 msec. Four buttons on the horizontal or vertical plane in front of the device were arranged to measure the time required to reciprocate motion. Fig. 1C and D shows the representative trajectories of a patient with SCA and an HC recorded using the device when the pen‐like part was moved between the yellow and green buttons. Every dot is a recording at 10‐msec intervals.

### Evaluation procedure and ataxia indices measured using the device

Participants were instructed to sit in a chair in front of the device and move a pen‐like part between the two buttons 9.5 times as quickly as possible. They performed the same tasks along four different routes (Fig. S1) in order, using their dominant and nondominant hands. To eliminate the effects of habituation and error, we removed the trajectory data of the first two and last reciprocating strokes. We also excluded the strokes that took the longest time. The average ataxia indices of the four routes of the dominant and nondominant hands were analyzed. We measured the trajectory length, time, and velocity of participants' upper limb movements. We also evaluated the variation coefficients of these parameters.

In addition, we developed a distortion index, which is a measurement to assess decomposition and dysmetria. We approximated the measured trajectories with a smooth nonlinear function using three different regression models: B‐spline regression, restricted cubic spline regression, and polynomial regression. The deviation of the observed trajectory from the approximate curve was calculated using the mean squared error (MSE) and the MSE values obtained from multiple trajectories for each patient was averaged and defined as the distortion index (Supplementary material).

### Motor function

We evaluated the severity of ataxia using functional scales. The SARA score ranged from 0 (no ataxia) to 40 (severe ataxia), and the ICARS score ranged from 0 (no ataxia) to 100 (severe ataxia). The severity of upper limb ataxia was evaluated using the upper limb SARA score, which consisted of three items (finger chase, nose–finger test, and fast alternating hand movements), with a range of 0–12, and the upper limb ICARS score, which consisted of five items (finger‐to‐nose test decomposition and dysmetria, finger‐to‐nose test intention tremor of the finger, finger–finger test, pronation–supination alternating movements, and drawing of the Archimedes spiral on a pre‐drawn pattern), with a range of 0–36. Participants also performed the nine‐hole pegboard test (9HPT) using a Rolyan 9HPT apparatus and a plastic one‐piece model using both hands.^18^ The test involved picking up nine pegs from a container and inserting them into the nine‐hole board, removing the pegs as quickly as possible, and placing them back into the container. The first test was performed with the dominant hand first and then the second with the nondominant hand, and the average time taken of both hands was the final score. We also evaluated non‐ataxia signs, such as pyramidal signs, weakness of limbs, involuntary movements, rigidity, and sensory symptoms in patients. Pyramidal signs were rated as present if hyperreflexia, extensor plantar reflex, or spasticity was positive. Involuntary movements included myoclonus, chorea, dyskinesia, and dystonia.

### Study design

Participants underwent motor functional assessments and evaluations using the device at each study visit. All participants were followed 12 months after the baseline evaluation to assess longitudinal changes. To evaluate test–retest reliability, consenting participants were assessed twice at an interval of 1–5 weeks.

### Statistical analyses

Statistical analyses were performed using the SPSS 25.0 J software (IBM Japan, Tokyo, Japan) or SAS version 9.4 (SAS Institute Inc., Cary, NC). All data are presented as means ± standard deviations (SD), unless otherwise stated. Chi‐squared tests and unpaired Student's *t*‐tests were used for comparisons of variables between patients with SCA and HCs. Pearson's correlation coefficients were used to identify correlations between parameters. A *p* value <0.05 was considered significant, and correlation coefficients (*r*) > 0.6 were interpreted as strong.^19^ Multivariate regression analysis, with a stepwise selection procedure, was used to evaluate the impact of age, sex, and genotype on each parameter. The stepping criteria employed for entry and removal were based on the significance level of the *F*‐value, which was set at 0.05. The equation was selected according to the highest multiple correlation coefficient. Test–retest reliability was assessed using intra‐class correlation coefficients (ICCs), specifically ICC (1, 1). ICC values <0.5 indicated poor reliability, values between 0.5 and 0.75 indicated moderate reliability, values between 0.75 and 0.9 indicated good reliability, and values greater than 0.9 indicated excellent reliability.^20^ The standard error of measurements (SEM) based on the test–retest parameter was calculated using the formula $\sqrt{\mathrm{SD}\left(\text{baseline}\right)2+\mathrm{SD}\left(\text{followup}\right)2}$/2 × $\sqrt{1-\mathrm{ICC}}$. Minimal detectable changes (MDCs) at the 95% confidence level (MDC_95_) was calculated using the formula 1.96 × $\sqrt{2}\;$× SEM. MDCs are defined as a change beyond measurement error and helps to provide a better interpretation of change.^21^ For the longitudinal analysis, the standardized response mean (SRM), which is the mean score change divided by the SD of the score change, was calculated as an index of the effect size for comparisons between parameters. To avoid underestimating the changes in parameters, the adjusted effect size (aES) was also calculated using the formula SRM × $\sqrt{2}\;$× $\sqrt{1-r}$, where *r* is Pearson's correlation coefficient.^22^ The aES was interpreted as trivial (aES < 0.20), small (aES ≥ 0.20 < 0.50), moderate (aES ≥ 0.50 < 0.80), or large (aES ≥ 0.80).^22^ Sample sizes were estimated by the observed parameter changes for a hypothetical intervention that should reduce progression rates by 10%–100% in steps of 10%, with a power of 0.8 and an α of 0.05.

### Data availability statement

Anonymized data of the current study will be made available to qualified investigators upon request.

## Results

### Participant characteristics and baseline measurements

**Figure 2:**
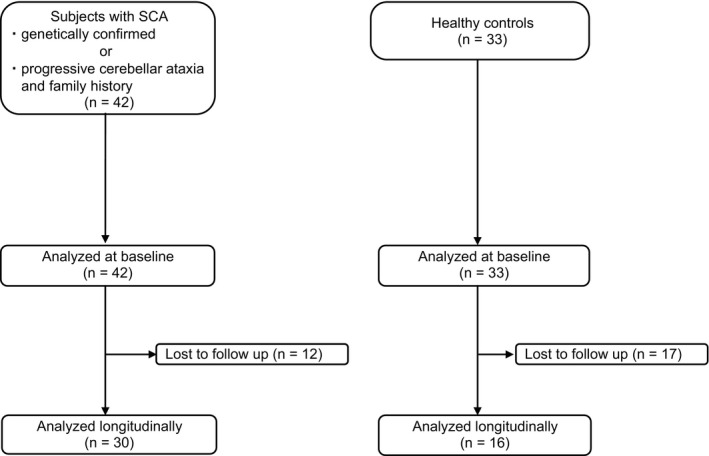
Flowchart of study population enrollment. Flowchart describing the process of study enrollment for patients with SCA and healthy controls. SCA, spinocerebellar ataxia.

A total of 42 patients with SCA and 33 HCs were recruited and evaluated at baseline. A total of 12 patients and 17 HCs were lost to follow‐up. A final total of 30 patients with SCA and 16 HCs were analyzed after 12 months of follow‐up (Fig. 2). Patients' baseline characteristics are shown in Table 1. The age at which patients first noticed ataxic symptoms was defined as the disease onset age. In our study population, the numbers of patients with SCA6 (10, 23.8%) and SCA31 (10, 23.8%) were higher than those in previous reports on the natural history of SCAs.^3^, ^23^, ^24^, ^25^ There were no statistically significant differences in mean age at the initial evaluation or sex ratios between the patients and HCs. Comparisons of the baseline characteristics between patients who underwent the 12‐month follow‐up and those lost to follow‐up are shown in Table S1. There was no significant difference in baseline characteristics between the two groups.

**Table 1:** Baseline characteristics, functional measurements, and ataxia indices of SCA and healthy subjects.

	SCA (*n* = 42)	HC (*n* = 33)	*p* Value*
Baseline characteristics			
Age at examination (years)	60.6 ± 10.7 (39–78)	59.6 ± 11.6 (40–82)	0.709
Gender, F/M	23/19	20/13	0.611
Disease duration (years)	9.2 ± 4.8 (0–21)	N.A.	N.A.
Genotypes SCA2/3/6/31/unknown	1/9/10/10/12	N.A.	N.A.
Pyramidal signs	20 (47.6%)	N.A	N.A
Weakness	12 (28.6%)	N.A	N.A
Involuntary movement	1 (2.4%)	N.A	N.A
Rigidity	3 (7.1%)	N.A	N.A
Sensory symptoms	13 (31.0%)	N.A	N.A
Functional measurements			
SARA total score	14.4 ± 5.8 (0–25.5)	0.4 ± 0.5 (0–2)	<0.001
SARA upper limb score	4.0 ± 1.7 (0–8.5)	0.4 ± 0.5 (0–2)	<0.001
ICARS total score	34.9 ± 14.0 (4–68)	1.3 ± 1.1 (0–4)	<0.001
ICARS upper limb score	8.5 ± 3.7 (0–16)	0.7 ± 1.0 (0–3)	<0.001
9‐hole peg test (sec)	45.8 ± 20.8 (23.1–116.0)	20.5 ± 3.0 (15.7–31.1)	<0.001
Ataxia indices measured using the device			
Trajectory length (mm)	3764 ± 294 (3277–4767)	3478 ± 182 (3119–3836)	<0.001
Time (sec)	13.7 ± 5.6 (6.8–32.7)	7.1 ± 1.1 (4.6–9.1)	<0.001
Velocity (mm/sec)	311 ± 93 (135–535)	510 ± 80 (368–761)	<0.001
Variation coefficient of the length	0.065 ± 0.016 (0.04–0.10)	0.045 ± 0.009 (0.03–0.07)	<0.001
Variation coefficient of the time	0.097 ± 0.032 (0.05–0.17)	0.060 ± 0.020 (0.03–0.11)	<0.001
Variation coefficient of the velocity	0.081 ± 0.021 (0.05–0.15)	0.053 ± 0.012 (0.03–0.08)	<0.001
Distortion index B‐spline	1.07 ± 0.33 (0.54–2.09)	0.55 ± 0.11 (0.40–0.92)	<0.001
Distortion index RCS	1.73 ± 0.46 (1.04–3.33)	0.96 ± 0.18 (0.68–1.62)	<0.001
Distortion index POLY	1.17 ± 0.35 (0.63–2.28)	0.61 ± 0.13 (0.44–1.16)	<0.001

SCA = spinocerebellar ataxia; HC = healthy control; N.A. = not available; SARA = scale for the assessment and rating of ataxia; ICARS = International Cooperative Ataxia Rating Scale; RCS = restricted cubic spline; POLY = polynomial regression.

Data represent mean ± standard deviation (range).

* Unpaired Student *t*‐test or Chi‐square test.

The mean SARA scores of the patients and HCs were similar to those reported in previous studies.^8^, ^12^, ^13^ For all functional measurements, there were significant differences between the patients with SCA and HCs, which indicated that these functional measurements discriminated patients with SCA of this stage from HCs. All indices measured using the pen‐like sensor device were also highly discriminative between the patients with SCA and HCs. The results in the dominant hand were similar to those in the nondominant hand (Table S2). Therefore, the average value of both hands was used for later analysis.

### Correlations of ataxia indices with clinical background and other measurements

We investigated the relationships between the ataxia indices measured using the device and age, disease duration, and functional scale scores to evaluate the validity of the measurements. For the ataxia indices, the average time required for the task, mean velocity, and distortion index in the regression models correlated significantly with not only disease duration, but also the functional scale scores (Table 2 and Fig. S4). There was no significant correlation between the ataxia index measures using the device and age at examination. Among the ataxia indices, the mean velocity showed the strongest correlation with the SARA and ICARS total scores, and the distortion index B‐spline showed the strongest correlation with the SARA and ICARS upper limb scores.

**Table 2:** Correlations of ataxia indices with disease‐related parameters.

	Age	Disease duration	SARA total score	SARA upper limb score	ICARS total score	ICARS upper limb score
9HPT	NS	0.360	0.711	0.614	0.712	0.672
Length	NS	NS	0.392	0.474	0.413	0.382
Time	NS	0.454	0.695	0.518	0.724	0.660
Velocity	NS	−0.471	−0.792	−0.563	−0.797	−0.718
Variation coefficient of the length	NS	NS	0.322	0.450	0.343	0.387
Variation coefficient of the time	NS	NS	0.348	0.463	0.384	0.470
Variation coefficient of the velocity	NS	NS	0.574	0.619	0.588	0.602
Distortion index B‐spline	NS	0.428	0.636	0.647	0.683	0.722
Distortion index RCS	NS	0.385	0.575	0.604	0.634	0.663
Distortion index POLY	NS	0.435	0.625	0.642	0.676	0.719

SARA = scale for the assessment and rating of ataxia; ICARS = International Cooperative Ataxia Rating Scale; 9HPT = 9‐hole peg test; RCS = restricted cubic spline; POLY = polynomial regression; NS = not significant.

Data represent Pearson correlation coefficient if *p* < 0.05, two sided.

### Covariates that affect ataxia indices

To evaluate the effects of age, sex, and genotype on ataxia indices in the patients with SCA, we performed a multivariate regression analysis. The stepwise multivariate linear regression analysis showed that only sex was independently correlated with the 9HPT score. For the ataxia indices measured using the device, none of the factors were correlated with any of the parameters.

### Reliability of ataxia indices measured using the device

To estimate the reliability of assessments using the device, six patients with SCA and one HC were examined twice with an interval of 22.1 ± 10.6 days. The ICCs for each ataxia index measured using the device ranged from 0.560 to 0.987, as shown in Table S3. The ICCs of all the indices, except for the variation coefficient of the velocity, were good or excellent.

### Longitudinal analyses of the functional measurements and ataxia indices

To evaluate the sensitivity of ataxia indices, we prospectively analyzed the longitudinal changes in functional measures in the patients with SCA and HCs over 12 months (Table 3 and 4). Among all the measures, only the distortion indices for all the regression models showed a statistically significant deterioration. The mean longitudinal changes of distortion index B‐spline and POLY were higher than MDC_95_ of them (Table S3), indicating that the longitudinal changes we detected were beyond measurement errors.

**Table 3:** Longitudinal analyses of functional measurements and indices in SCA patients.

	Baseline 0 W (SD)	Follow‐up 48 W (SD)	Mean longitudinal change (SD)	*p* Value*	SRM	aES
SARA total score	14.3 (5.6)	15.1 (5.4)	0.8 (2.6)	0.115	0.30	0.14
SARA upper limb score	4.0 (1.7)	4.0 (1.6)	−0.1 (1.1)	0.687	−0.07	−0.05
ICARS total score	34.1 (13.4)	36.5 (12.8)	2.4 (5.7)	0.029	0.42	0.18
ICARS upper limb score	8.6 (3.5)	8.7 (3.4)	0.1 (3.0)	0.855	0.03	0.03
9‐hole peg test (sec)	44.5 (20.1)	44.5 (19.6)	0.0 (11.9)	0.988	0.00	0.00
Trajectory length (mm)	3716 (280)	3821 (392)	104 (284)	0.054	0.37	0.29
Time (sec)	13.2 (5.1)	13.2 (5.0)	0.05 (2.2)	0.900	0.02	0.01
Velocity (mm/sec)	316 (93)	323 (96)	7 (47)	0.455	0.14	0.07
Variation coefficient of the length	0.066 (0.015)	0.073 (0.023)	0.008 (0.021)	0.054	0.37	0.37
Variation coefficient of the time	0.095 (0.030)	0.100 (0.040)	0.005 (0.027)	0.294	0.20	0.14
Variation coefficient of the velocity	0.080 (0.018)	0.085 (0.020)	0.005 (0.017)	0.138	0.28	0.25
Distortion index B‐spline	1.04 (0.30)	1.21 (0.41)	0.17 (0.30)	0.004	0.57	0.45
Distortion index RCS	1.67 (0.43)	1.91 (0.64)	0.23 (0.51)	0.018	0.46	0.41
Distortion index POLY	1.14 (0.32)	1.30 (0.44)	0.16 (0.30)	0.008	0.52	0.39

SCA = spinocerebellar ataxia; SARA = scale for the assessment and rating of ataxia; ICARS = International Cooperative Ataxia Rating Scale; RCS = restricted cubic spline; POLY = polynomial regression; SD = standard deviation, SRM = standardized response mean; aES = adjusted effect size.

Data represent mean (standard deviation).

* Paired *t*‐test.

**Table 4:** Longitudinal analyses of functional measurements and indices in healthy controls.

	Baseline 0 W (SD)	Follow‐up 48 W (SD)	Mean longitudinal change (SD)	*p* Value*	SRM	aES
SARA total score	0.5 (0.6)	0.7 (0.6)	0.2 (0.5)	0.188	0.34	0.31
SARA upper limb score	0.5 (0.6)	0.6 (0.6)	0.1 (0.5)	0.423	0.21	0.15
ICARS total score	1.8 (1.1)	2.3 (1.4)	0.5 (1.4)	0.178	0.35	0.39
ICARS upper limb score	1.1 (1.2)	0.8 (0.9)	−0.3 (1.1)	0.289	−0.27	−0.29
9‐hole peg test (sec)	21.5 (3.4)	21.4 (3.1)	−0.1 (2.1)	0.871	−0.04	−0.03
Trajectory length (mm)	3546 (167)	3474 (167)	−72 (115)	0.024	−0.63	−0.43
Time (sec)	7.5 (1.0)	6.8 (0.6)	−0.7 (0.8)	0.002	−0.94	−0.82
Velocity (mm/sec)	487 (72)	523 (51)	36 (46)	0.007	0.78	0.53
Variation coefficient of the length	0.046 (0.008)	0.044 (0.007)	−0.002 (0.008)	0.413	−0.21	−0.22
Variation coefficient of the time	0.062 (0.023)	0.057 (0.017)	−0.005 (0.019)	0.306	−0.26	−0.24
Variation coefficient of the velocity	0.056 (0.013)	0.048 (0.011)	−0.008 (0.012)	0.023	−0.63	−0.61
Distortion index B‐spline	0.58 (0.08)	0.57 (0.08)	−0.01 (0.06)	0.395	−0.22	−0.16
Distortion index RCS	0.98 (0.15)	0.93 (0.15)	−0.05 (0.14)	0.172	−0.36	−0.32
Distortion index POLY	0.64 (0.08)	0.63 (0.10)	−0.01 (0.07)	0.596	−0.14	−0.10

SCA = spinocerebellar ataxia; SARA = scale for the assessment and rating of ataxia; ICARS = International Cooperative Ataxia Rating Scale; RCS = restricted cubic spline; POLY = polynomial regression; SD = standard deviation, SRM = standardized response mean; aES = adjusted effect size.

Data represent mean (standard deviation).

* Paired *t*‐test.

The total SARA and ICARS scores showed a trend increase in patients with SCA, as reported previously.^3^, ^26^ However, the SARA and ICARS upper limb scores, and 9HPT were largely unchanged over the 12 months. HCs showed a mild but statistically significant improvement in time, length, velocity, and variation coefficient of the velocity, which indicated a learning effect; such a longitudinal change was not observed in the SCA group. HCs showed no changes in distortion indices for all the regression models, which suggested that distortion indices were less affected by the learning effect.

**Figure 3:**
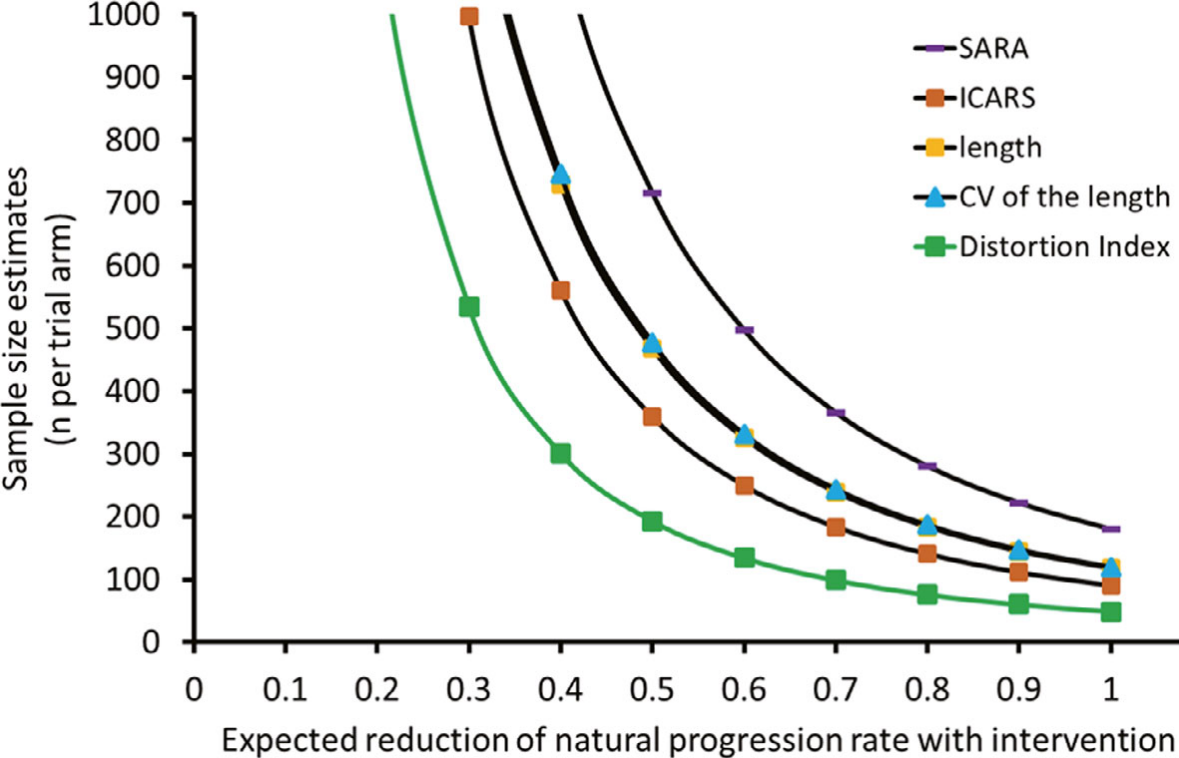
Sample size estimation. Estimated sample size per arm for a two‐arm interventional clinical trials that aim to reduce natural progression rates by 10%–100% based on the observed variability of the chronological progression rates found in the present study (power 0.80, *α* 0.05). SARA, scale for the assessment and rating of ataxia; ICARS, International Cooperative Ataxia Rating Scale; CV, coefficient of variation. [Colour figure can be viewed at wileyonlinelibrary.com]

The aES of the distortion index B‐spline was the largest of all the functional measures, which indicated that this index requires the smallest sample size for clinical trials and is the most sensitive to disease progression. The sample size estimation based on the longitudinal analysis showed that the distortion index B‐spline would require the smallest sample size (Fig. 3). A total of 193 (distortion index B‐spline) and 359 (ICARS) patients per arm would be required to detect a 50% reduction in disease progression in a two‐arm trial within 1 year using the functional parameters, which suggests that the distortion index is a sensitive clinical measure.

### Comparison of longitudinal changes in functional measurements and indices for phenotype, stage, and genotype

To assess the effects of non‐ataxia symptoms on the longitudinal changes in ataxia indices, we divided SCA patients into two groups based on the presence/absence of non‐ataxia signs (Table S4). In both groups, only distortion index B‐spline and POLY showed a statistically significant deterioration, and the aES values of them were large. Mean longitudinal changes in distortion indices were larger in SCA patients without non‐ataxia symptoms than in those with non‐ataxia symptoms. These results suggest that non‐ataxia symptoms possibly obscure the deterioration of distortion indices.

Next, to investigate the effects of disease severity on the longitudinal changes of indices, we classified SCA patients into those at an early stage with their SARA total score being ≤13.5, the median value of the total population, or those at a late stage with SARA >13.5 (Table S5). At an early stage, SARA total score and ICARS total score significantly worsened over 12 months, but at a late stage deterioration of these scores attenuated. On the other hand, the distortion index B‐spline showed deterioration regardless of the disease stage.

Lastly, we assessed the usefulness of the distortion indices in each genotype (Tables S6–S8). In SCA6 and SCA31, the aES of the distortion index B‐spline was the largest among all the functional measures. The aES values of the distortion indices were smaller, and their mean longitudinal changes were lower, in SCA3 than in SCA6 and SCA31.

## Discussion

In this study, we developed the device to evaluate the upper limb movements of patients with SCA. Significant differences were found between patients with SCA and HCs across all parameters measured using this device. The parameters were also correlated with the upper limb scores of the SARA and ICARS. Furthermore, distortion indices, which depict the deviation of the observed trajectory from the approximate curve, showed longitudinal deterioration, whereas other measures, including the SARA, failed to demonstrate such changes. The longitudinal deterioration of distortion indices was not observed in HCs, which suggested that the indices detect motor functional changes associated with SCA disease progression. In addition, distortion indices, especially distortion index B‐spline, showed longitudinal deterioration regardless of the disease severity, but the degree of deterioration was diminished in patients with non‐ataxic symptoms.

Quantitative biomarkers that monitor disease severity are essential to estimate the efficacy of treatments tested in clinical trials.^27^, ^28^, ^29^ In general, satisfactory biomarkers should possess good validity, reliability, and sensitivity to longitudinal change.^30^ To date, semiquantitative rating scales, such as the SARA or ICARS, are used widely to evaluate the severity of ataxia in clinical trials; however, these scales are not suitable for detecting small changes in ataxia severity over a short period.^31^, ^32^ In addition, the inter‐rater reliability of these scales is inadequate, especially for the assessment of upper limb ataxia.^8^, ^11^ Our device‐aided measurement provided a more effective assessment of longitudinal change over 1 year than that of the functional scales and 9HPT.

Among the ataxia indices measured using the device, the distortion index B‐spline, which indicates dysmetria and decomposition, had the strongest correlation with the upper limb scores of the SARA and ICARS and was the most sensitive to the longitudinal change of the disease. Moreover, the distortion index B‐spline had excellent reliability. In contrast, the other indices did not detect significant longitudinal changes over 12 months, although the mean velocity was strongly correlated with the SARA and ICARS total scores. These results are consistent with previous reports that suggest the degree of deviation from the ideal trajectory was more important than the time spent for upper limb movement.^33^ It has been reported that when the trajectory deviation and time spent were measured simultaneously along two dimensions of two consecutive triangles displayed on a digitizer, the area of the deviation between the actual trajectory and the triangles contributed more to the discrimination of upper limb ataxia severity than it did to time.^33^ Another study suggested that the gap between the Archimedean spiral template and the drawn spiral measured using the Image J software is related to the severity of ataxia and cerebellar volume.^15^ Furthermore, a study that used a Kinect sensor demonstrated that compared with the fluctuation of the index finger, the average speed of the index finger in the nose–finger test was correlated more strongly with the SARA total score.^17^

The low sensitivity of velocity, yet high sensitivity of the distortion index B‐spline may be attributed to the measurement methods used. Specifically, the pen‐like tool we used may be more sensitive to the distance than to the velocity of hand movement; a previous study using a three‐dimensional movement analyzer suggested that the use of a pen detects disease‐associated changes in distance more sensitively than those in velocity.^34^ Alternatively, our instruction to the participants to move their upper limbs as quickly as possible may have led participants to move their hands quickly at the expense of a precise trajectory.

In contrast to previous studies, our results showed no significant change in the 9HPT score in 12 months. In a previous study on patients with SCA, 9HPT performance deteriorated significantly over time.^24^ This discrepancy may be due to the differences in genetic backgrounds between our patients and those of previous studies. It is generally accepted that the disease progression of SCA6 and SCA31 is slower than that of SCA1, SCA2, and SCA3.^10^, ^35^ As mentioned above, in our study population, the numbers of patients with SCA6 and SCA31 were higher than those in previous reports, which may reflect the high prevalence of SCA6 and SCA31 in Japan.^36^, ^37^

The longitudinal change of distortion indices was smaller in SCA3 compared to SCA6 and SCA31. There are two possible explanations for this result. First, non‐ataxia symptoms may diminish the longitudinal change of distortion indices. As previously reported,^10^, ^38^ the frequency of non‐ataxia symptoms was higher in SCA3 than in SCA6 and SCA31 in our study. Given that the longitudinal changes in distortion indices tended to be lower in SCA patients with non‐ataxia symptoms in the present study, it is possible that non‐ataxia symptoms obscure the deterioration of distortion indices. Second possible reason is the difference in the components of ataxia among diseases. In a study on the individual SARA items in various types of SCA, patients with SCA3 had better scores of item 5 (finger chase) and item 6 (nose–finger test) than patients with SCA1, SCA2, or SCA6, possibly due to differences in pattern of degeneration.^39^ As our device evaluated hand movement, the difference in these items among diseases may be reflected in the longitudinal change of distortion indices.

Although our results demonstrated that the distortion index was a valid, reliable, and sensitive biomarker of SCA, there are several limitations to this study. First, our small patient sample limits the interpretation of the results. Second, we evaluated trajectory deviation by calculating the difference between the approximate and observed curves, which is a method that is yet to be verified. The validity of our method needs confirmation in further studies. Third, cognitive function was not assessed in our study. Previous studies have reported correlations between cognitive impairment, 9HPT score, and walking capacity.^12^, ^40^ Recently, it was demonstrated that patients with SCA may have cognitive impairment called cerebellar cognitive affective syndrome (CCAS), which is characterized by disturbances in executive function, impaired spatial cognition, personality changes, and linguistic difficulties.^41^ In future studies, the effects of CCAS on functional measurements should be analyzed.

In summary, we developed a new method to evaluate upper limb ataxia in patients with SCA. This study indicated that the distortion index is the most reliable biomarker of the severity of upper limb ataxia, and it would require a smaller sample size for use in clinical trials than would the existing scales of the SARA and ICRAS. Thus, this measure may be a useful outcome measure for clinical trials. Future studies to confirm our results are needed to establish a much‐needed endpoint for clinical trials for SCA.

## Author Contributions

Drs. Kishimoto, Hashizume, Nagano, Fujimoto, and Katsuno conceived and designed the study. Drs. Kishimoto, Hashizume, Yamada, Ito, and Torii contributed to the data acquisition. Drs. Kishimoto, Hashizume, Imai, and Nakatochi contributed to the statistical analysis and interpretation of the data. Dr. Kishimoto drafted the manuscript. Drs. Hashizume and Katsuno did the critical revision of the manuscript for important intellectual content. Drs. Hashizume and Katsuno had full access to all the data in the study and take responsibility for the integrity of the data and the accuracy of the data analysis.

## Conflict of Interest

The authors declare no conflict of interest associated with this manuscript.

## Supporting information


**Figure S1.** Evaluation procedure. Participants gripped the pen‐like tool and moved it between the two buttons in the order of yellow–green, red–pink, yellow–red, and pink–green. At first the task was performed with dominant hand and next the same task was performed with nondominant hand.
**Figure S2.** Calculation of distortion index. For each trajectory, each of *x*, *y*, and *z* was fitted by *d* using smooth nonlinear functions such as B‐spline regression, restricted cubic spline regression, and polynomial regression and the MSE was calculated between actual values and estimated values. As a result 336 MSEs were obtained per person. Three hundred and thirty‐six MSEs were averaged and rooted to obtain the distortion index. MSE, mean squared error.
**Figure S3.** Setting optimal parameters for fitting of the nonlinear model. Image of the deviation for each parameter of a trajectory of SCA005 yellow–green _z_inboound_nondominant hand (A). AUC of the deviation to discriminate patients with SCA from HCs and parameter for fitting of the nonlinear model (B). SCA, spinocerebellar ataxia; MSE, mean squared error; AUC, area under the curve; HCs, healthy controls.
**Figure S4.** Correlations between each functional measurement and ataxia index and disease duration, SARA total score and SARA upper limb score in patients with SCA. Correlations between nine‐hole peg test and disease severity‐related parameters: disease duration, SARA total score, and SARA upper limb score (A–C). Correlations between velocity and disease severity‐related parameters (D–F). Correlations between distortion index B‐spline and disease severity‐related parameters (G–I). Correlations between distortion index restricted cubic spline regression (RCS) and disease severity‐related parameters (J–L). Correlations between distortion index polynomial regression (POLY) and the disease severity‐related parameters (M–O). Significant correlation coefficients and *p* values are annotated. SCA, spinocerebellar ataxia; SARA, Scale for the Assessment and Rating of Ataxia.
**Table S1.** Baseline characteristics between patients with SCA with and without follow‐up.
**Table S2.** Ataxia indices in each hand of SCA and healthy subjects.
**Table S3.** Reliability and minimal detectable changes for the movement indices of the device.
**Table S4.** Longitudinal analyses of functional measurements and indices in SCA patients with and without non‐ataxia symptoms.
**Table S5.** Longitudinal analyses of functional measurements and indices in SCA patients at early and late stages.
**Table 6.** Longitudinal analyses of functional measurements and indices in SCA3 patients (*n* = 7).
**Table S7.** Longitudinal analyses of functional measurements and indices in SCA6 patients (*n* = 6).
**Table S8.** Longitudinal analyses of functional measurements and indices in SCA31 patients (*n* = 8).
**Supplemental methods.** Methods for evaluation procedure, data preprocessing, calculation of the distortion index, and statistical analyses.
**Supplemental References.** Reference for statistical analysis.
