# Supplementary material for: Quantitative evaluation of upper limb ataxia in spinocerebellar ataxias
Source: Ann Clin Transl Neurol. 2022 Mar 15;9(4):529–39. doi: 10.1002/acn3.51528 (PMC8994984; doi:10.1002/acn3.51528)
Supplement: Supplementary file 1 — Figure S1. Evaluation procedure. Participants gripped the pen‐like tool and moved it between the two buttons in the order of yellow–green, red–pink, yellow–red, and pink–green. At first the task was performed with dominant hand and next the same task was performed with nondominant hand. Figure S2. Calculation of distortion index. For each trajectory, each of x, y, and z was fitted by d using smooth nonlinear functions such as B‐spline regression, restricted cubic spline regression, and polynomial regression and the MSE was calculated between actual values and estimated values. As a result 336 MSEs were obtained per person. Three hundred and thirty‐six MSEs were averaged and rooted to obtain the distortion index. MSE, mean squared error. Figure S3. Setting optimal parameters for fitting of the nonlinear model. Image of the deviation for each parameter of a trajectory of SCA005 yellow–green _z_inboound_nondominant hand (A). AUC of the deviation to discriminate patients with SCA from HCs and parameter for fitting of the nonlinear model (B). SCA, spinocerebellar ataxia; MSE, mean squared error; AUC, area under the curve; HCs, healthy controls. Figure S4. Correlations between each functional measurement and ataxia index and disease duration, SARA total score and SARA upper limb score in patients with SCA. Correlations between nine‐hole peg test and disease severity‐related parameters: disease duration, SARA total score, and SARA upper limb score (A–C). Correlations between velocity and disease severity‐related parameters (D–F). Correlations between distortion index B‐spline and disease severity‐related parameters (G–I). Correlations between distortion index restricted cubic spline regression (RCS) and disease severity‐related parameters (J–L). Correlations between distortion index polynomial regression (POLY) and the disease severity‐related parameters (M–O). Significant correlation coefficients and p values are annotated. SCA, spinocerebellar ataxia; SARA, Scale for the [file ACN3-9-529-s001.docx]

**Supplemental data**

**Quantitative evaluation of upper limb ataxia in spinocerebellar ataxias**

Yoshiyuki Kishimoto, MD, Atsushi Hashizume, MD, PhD, Yuta Imai, Masahiro Nakatochi, PhD, Shinichiro Yamada, MD, PhD, Daisuke Ito, MD, PhD, Ryota Torii, MD, Yoshitaka Nagano, PhD, Hideo Fujimoto, PhD, and Masahisa Katsuno, MD, PhD

**Contents:**

Supplemental Methods

Supplemental References

Four Supplemental Figures (supplementary figure 1–4)

Eight Supplemental Tables (supplementary table 1–8)

**Supplemental methods**

**Evaluation procedure**

Participants were instructed to sit in a chair in front of the device so that the centerline of their body was in line with the center of the device. Next, participants gripped the pen-like tool with their dominant hand and moved it back and forth as quickly as possible between the two buttons in the order of yellow–green, red–pink, yellow–red, and pink–green (Supplementary Figure 1). The same task was then performed with their nondominant hand. They were permitted to practice it once before testing. When participants missed hitting the button, they were asked to perform the task again.

**Data preprocessing**

The three-dimensional trajectory data were acquired from 42 patients and 33 HCs using the novel device. Trajectories of four different routes were measured for each participant. The trajectory data per route were recorded in three-dimensional coordinates of *x*, *y*, and *z* every 10 ms for 9.5 round trips (19 trajectories in total). The number of points constituting a single trajectory varied between trajectories: the minimum number of points constituting a single trajectory was 29, the maximum was 364, and the average was 79.

The trajectory data underwent the following preprocessing. First, to unify the number of points constituting trajectories, a linear interpolation method was applied to each of the *x*, *y*, and *z* axes data of each trajectory based on the distance traveled in three dimensions, and the number of constituent points of each trajectory data was unified to 100 points. Next, to eliminate the effect of unfamiliarity at the time of the first measurement and familiarity at the end of the measurement along the same route, we removed the trajectory data of the first round trip and the last one-way trip for each route. As a result, eight round trips, consisting of 16 trajectories remained. To avoid inaccurate trajectory data because of missed switch presses, trajectory data of the eight round trips with the longest time spent were also removed, which left seven round trips (14 trajectories) per route. With the above preprocessing, seven round trips × 2 (outbound and inbound) × 4 types of routes × hand used for measurement (dominant and nondominant hands) × three-dimensional coordinates (*x*, *y*, and *z*) = 336 trajectory data points per person, were used for further analysis.

**Calculation of the distortion index**

The basic concept of the distortion index developed in this study is shown in Supplementary Figure 2. The score was evaluated for a single trajectory consisting of three-dimensional coordinates, which was decomposed into the relationship between the total distance (*d*) traveled from the starting point at each point and each of the *x*, *y*, and *z* coordinates. Following the preprocessing described above, the total distance traveled, *d*, was coded as (1, 2, … 100). We developed the distortion index based on the assumption that the relationship between the total distance traveled, *d*, and each of the *x*, *y*, and *z* coordinates can be described as a smooth trajectory in HCs and a complex trajectory in patients. First, for each trajectory, *x*, *y*, and *z* were fitted to *d* using smooth nonlinear functions, such as polynomial regression. Next, the squared error $\text{e}_{\text{i}}^{\text{2}}$ was calculated between the actual values of the *x*, *y*, and *z* coordinates at *d = i* and the estimates of *x*, *y*, and *z* obtained by substituting *d = i* into the model. Third, the MSE = $\text{Σ}_{\text{i=1}}^{\text{100}}\text{e}_{\text{i}}^{\text{2}}\text{/100}$ was obtained by averaging the $\text{e}_{\text{i}}^{\text{2}}$ values for all 100 points comprising the trajectory. In Supplementary Figure 2, the MSE is low for HCs because the $\text{e}_{\text{i}}^{\text{2}}$ between the modeled curve and the actual coordinates were small, whereas for patients, the MSE is large because some of the $\text{e}_{\text{i}}^{\text{2}}$ values were large. For the distortion index of each subject, 336 different MSEs were calculated based on the 336 trajectories obtained from each subject, and the mean of these was used as the distortion index. A low distortion index indicated a HC and a high distortion index indicated a patient. Patients with a higher distortion index were also expected to have a greater severity of the disease.

The data of 336 trajectories per subject were fitted to nonlinear models, such as B-spline regression, restricted cubic spline regression, and polynomial regression. The fitting of the nonlinear model was performed for each of the 336 trajectories. First, three-dimensional trajectory data were divided into the three components of *x*, *y*, and *z*. Then, based on the data of each of the three components of *x*, *y*, and *z*, and the total distance traveled, *d*, the data were applied to three types of nonlinear models: B-spline regression, restricted cubic spline regression, and polynomial regression. The dependent variable was the value of the *x*, *y*, or *z* coordinate. The independent variable was the distance traveled, *d*, from the starting point to each point. Next, $\text{e}_{\text{i}}^{\text{2}}$ was calculated as the difference between the estimated value from the nonlinear function and the actual coordinates. The MSE of the $\text{e}_{\text{i}}^{\text{2}}$ values obtained from the 100 points was calculated. This process was performed for each of the *x*, *y*, and *z* coordinates of the outward and inward trajectories of the seven round trips to obtain the MSE. The 3 (*x*, *y*, and *z*) × 7 × 2 (outbound and inbound) × 4 (routes) × 2 (dominant and nondominant hands) MSEs were then averaged and square rooted to obtain a distortion index for a single trajectory condition. The distortion index will have a value close to zero if the actual trajectory is a smooth curve and a higher value if it is not.

In the spline and polynomial regression models used in this study, there was a parameter that controlled the complexity of the model. For B-spline and restricted cubic spline regression, it was the number of knots; and for polynomial regression, it was the degree of the polynomial. An example of the parameters and curve estimations are shown in the upper part of Supplementary Figure 3, which shows a specific trajectory. The larger the parameter value, the more complex the curve that can be fitted. To develop an optimal distortion index, we fitted the model with several candidate parameters and calculated the MSE. We adopted the parameter with the highest discriminatory power between patients and HCs, as shown in the lower panel of Supplementary Figure 3. Discriminative ability was assessed based on the area under the curve by conducting a receiver operating characteristic (ROC) analysis. The same procedure was carried out for all combinations of routes, hand dominance, and *x*, *y*, and *z* coordinates to determine the optimal parameters for each model.

To calculate the final distortion index for each sample, the trajectory data were refitted to the model using the optimal parameters, and the distortion index was calculated. As a result, 336 distortion indices per model were calculated for each sample.

**Statistical analyses**

These scores were averaged for each sample and used as the final distortion indices. The distortion indices calculated were used to evaluate the differences between HCs and patients. Student’s *t*-tests were used for the calculations.

A paired *t*-test was used to evaluate the change in distortion index in patients at the time of the measurement to 48 weeks after the measurement.

All processing was performed using R (version 4.0.2,<https://www.r-project.org/>). The rms package was used for the restricted cubic spline regression, and the splines package was used for the B-spline regression. The pROC package was used for the ROC analysis^1^. The significance level was set at 5%.

**Supplemental References**

e1. Robin X, Turck N, Hainard A, et al. pROC: An open-source package for R and S+ to analyze and compare ROC curves. *BMC Bioinformatics* 2011;12:1-17.

**Supplementary Figure 1.** Evaluation procedure

**
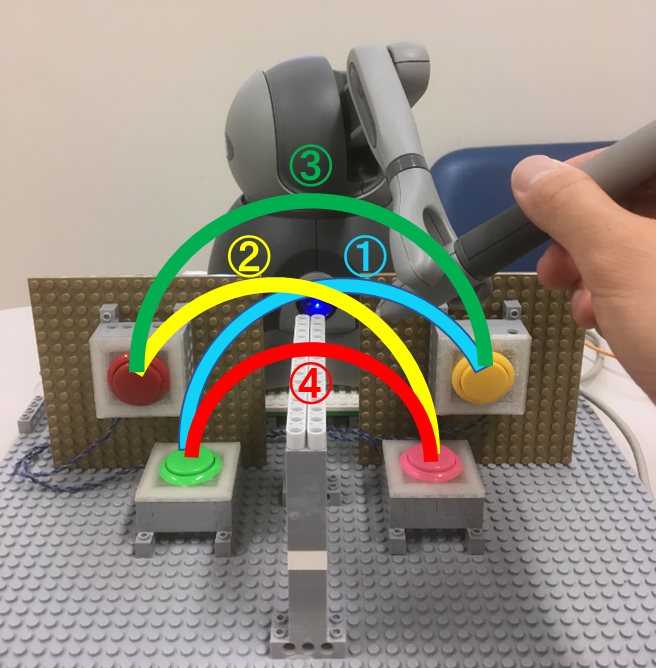
**

Participants gripped the pen-like tool and moved it between the two buttons in the order of yellow–green, red–pink, yellow–red, and pink–green. At first the task was performed with dominant hand and next the same task was performed with nondominant hand.

**Supplementary Figure 2.** Calculation of distortion index


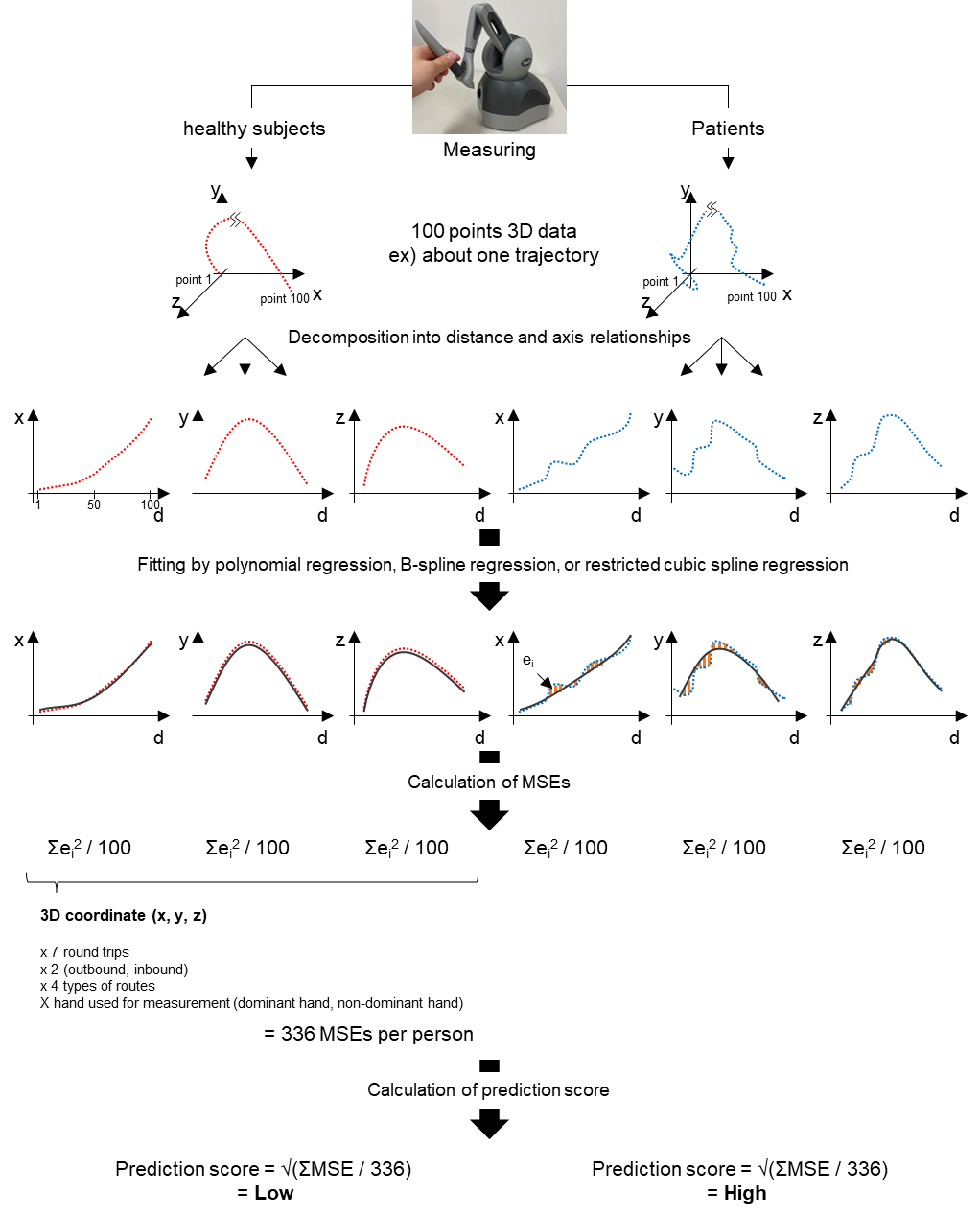


For each trajectory, each of *x*, *y*, and *z* was fitted by *d* using smooth nonlinear functions such as B-spline regression, restricted cubic spline regression, and polynomial regression and the MSE was calculated between actual values and estimated values. As a result 336 MSEs were obtained per person. 336 MSEs were averaged and rooted to obtain the distortion index. MSE, mean squared error.


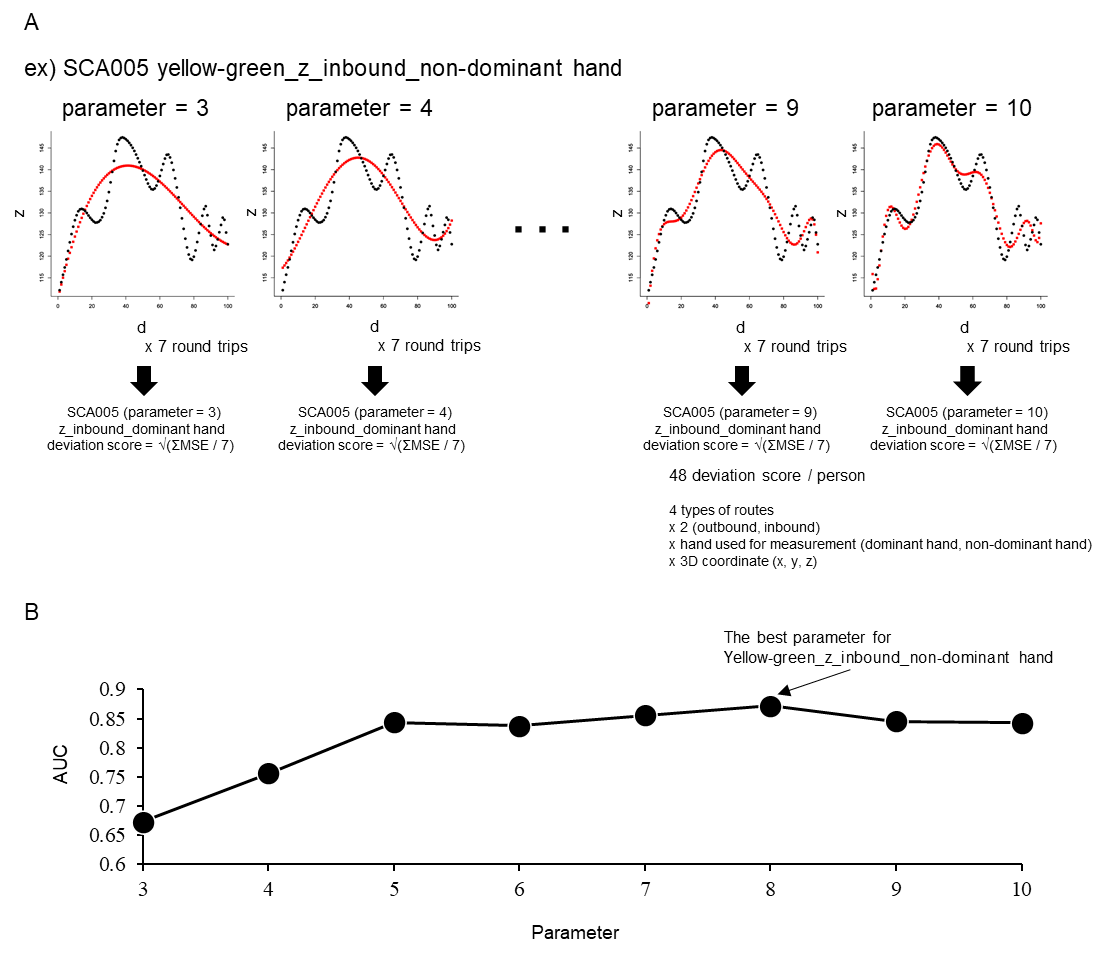
**Supplementary Figure 3.** Setting optimal parameters for fitting of the nonlinear model.

Image of the deviation for each parameter of a trajectory of SCA005 yellow-green _z_inboound_non-dominant hand (A). AUC of the deviation to discriminate patients with SCA from HCs and parameter for fitting of the nonlinear model (B). SCA, spinocerebellar ataxia; MSE, mean squared error; AUC, area under the curve; HCs, healthy controls.


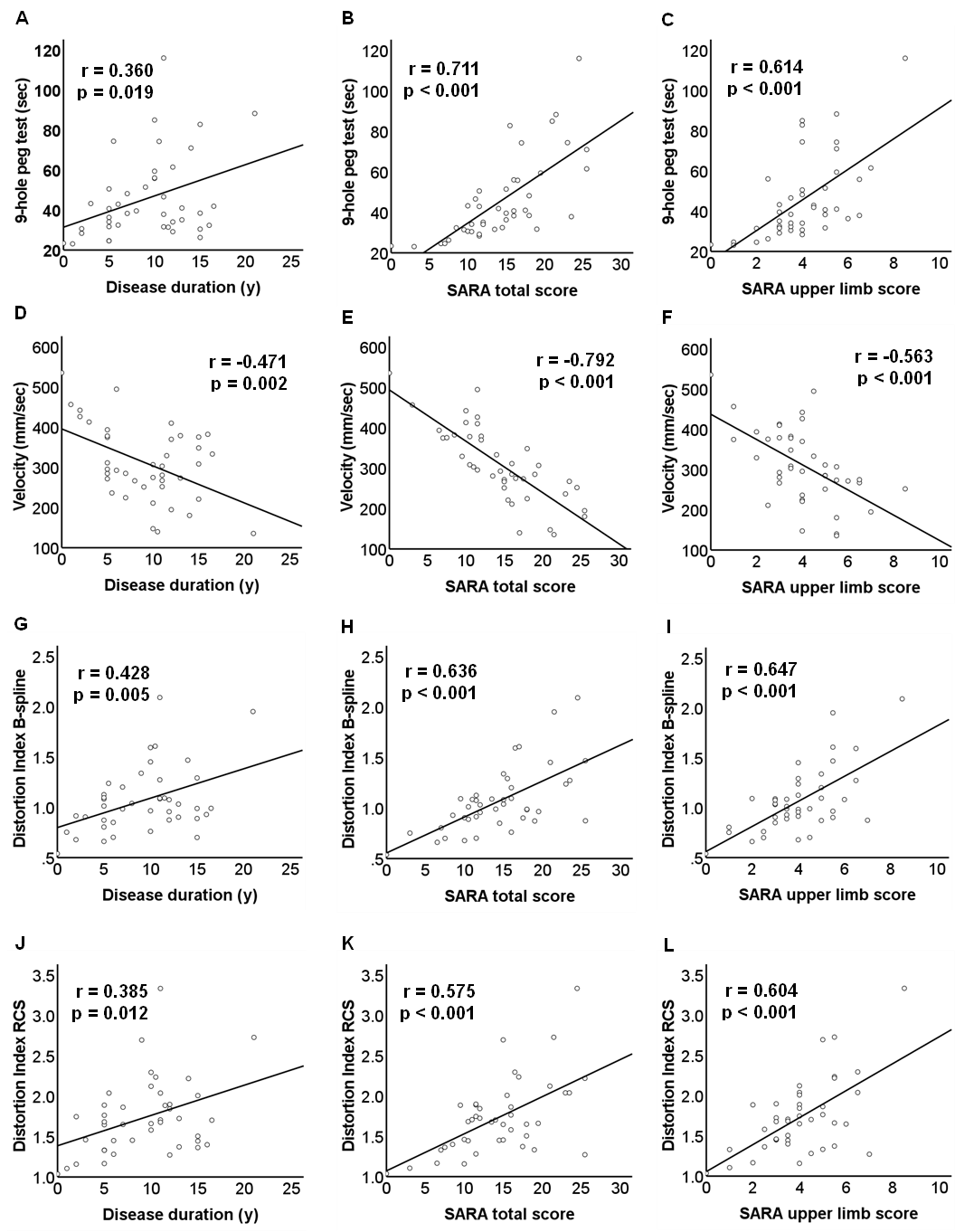
**Supplementary Figure 4.** Correlations between each functional measurement and ataxia index and disease duration, SARA total score and SARA upper limb score in patients with SCA.

**
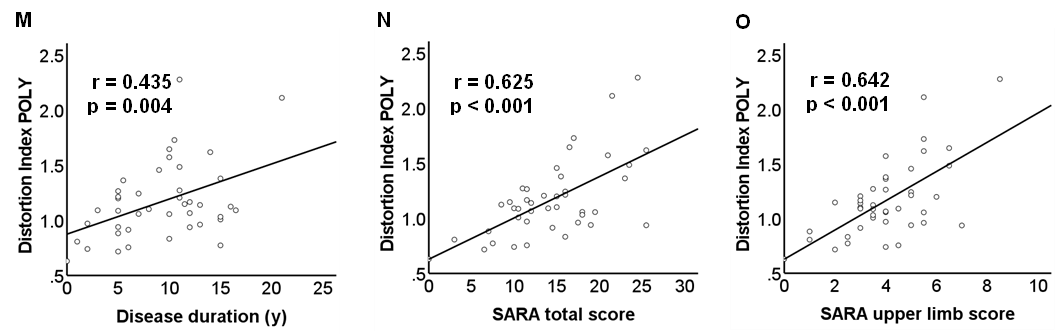
**

Correlations between 9-hole peg test and disease severity-related parameters: disease duration, SARA total score, and SARA upper limb score (A-C). Correlations between velocity and disease severity-related parameters (D-F). Correlations between distortion index B-spline and disease severity-related parameters (G-I). Correlations between distortion index restricted cubic spline regression (RCS) and disease severity-related parameters (J-L). Correlations between distortion index polynomial regression (POLY) and the disease severity-related parameters (M-O). Significant correlation coefficients and *p* values are annotated. SCA, spinocerebellar ataxia; SARA, Scale for the Assessment and Rating of Ataxia.

**Supplementary Table 1.** Baseline characteristics between patients with SCA with and without follow-up

|  | **SCA analyzed longitudinally**  **(n = 30)** | **SCA lost to follow up (n = 12)** | ***p* Value*** |
| --- | --- | --- | --- |
| **Age at examination (years)** | 62.4 ± 9.8 (40-78) | 55.9 ± 11.8 (39-76) | 0.073 |
| **Gender, F/M** | 18/12 | 5/7 | 0.281 |
| **Disease duration (years)** | 9.3 ± 4.4 (0-16.5) | 9.1 ± 5.9 (1-21) | 0.940 |
| **Genotypes SCA2/3/6/31/unknown** | 1/7/6/8/8 | 0/2/4/2/4 | N.A. |
| **SARA total score** | 14.3 ± 5.6 (0-25.5) | 14.6 ± 6.5 (3-23.5) | 0.873 |
| **SARA upper limb score** | 4.0 ± 1.7 (0-8.5) | 4.0 ± 1.8 (1-6.5) | 0.989 |
| **ICARS total score** | 34.1 ± 13.4 (4-68) | 36.7 ± 15.8 (12-60) | 0.602 |
| **ICARS upper limb score** | 8.6 ± 3.5 (0-16) | 8.2 ± 4.2 (1-14) | 0.736 |

Abbreviations: SCA = spinocerebellar ataxia; SARA = Scale for the Assessment and Rating of Ataxia; ICARS = International Cooperative Ataxia Rating Scale; N.A. = not available.

Data represent mean ± standard deviation (range). *Unpaired Student *t* test or Chi-square test.

**Supplementary Table 2.** Ataxia indices in each hand of SCA and healthy subjects

|  | | **SCA (n = 42)** | **HC (n = 33)** | ***p* Value*** |
| --- | --- | --- | --- | --- |
| **Ataxia indices measured using the device** | |  |  |  |
| **Dominant hand** | |  |  |  |
|  | **Trajectory length (mm)** | 3672 ± 320 (3065-4833) | 3431 ± 216 (3058-4023) | <0.001 |
|  | **Time (sec)** | 12.8 ± 5.3 (6.6-31.8) | 6.6 ± 1.1 (4.1-8.6) | <0.001 |
|  | **Velocity (mm/sec)** | 325 ± 101 (138-556) | 538 ± 92 (404-827) | <0.001 |
|  | **Variation coefficient of the length** | 0.059 ± 0.014 (0.03-0.09) | 0.041 ± 0.010 (0.03-0.07) | <0.001 |
|  | **Variation coefficient of the time** | 0.086 ± 0.031 (0.04-0.16) | 0.052 ± 0.019 (0.03-0.13) | <0.001 |
|  | **Variation coefficient of the velocity** | 0.075 ± 0.023 (0.03-0.15) | 0.048 ± 0.012 (0.03-0.09) | <0.001 |
|  | **Distortion index B-spline** | 1.07 ± 0.38 (0.52-2.36) | 0.52 ± 0.12 (0.35-0.85) | <0.001 |
|  | **Distortion index RCS** | 1.97 ± 0.58 (1.16-4.24) | 1.13 ± 0.23 (0.75-1.85) | <0.001 |
|  | **Distortion index POLY** | 1.18 ± 0.42 (0.65-2.61) | 0.60 ± 0.14 (0.38-1.08) | <0.001 |
| **Nondominant hand** | |  |  |  |
|  | **Trajectory length (mm)** | 3857 ± 331 (3323-4701) | 3525 ± 193 (3148-3882) | <0.001 |
|  | **Time (sec)** | 14.7 ± 6.1 (7.0-33.6) | 7.5 ± 1.2 (5.1-9.9) | <0.001 |
|  | **Velocity (mm/sec)** | 296 ± 89 (128-514) | 482 ± 76 (332-695) | <0.001 |
|  | **Variation coefficient of the length** | 0.072 ± 0.026 (0.03-0.13) | 0.049 ± 0.013 (0.03-0.10) | <0.001 |
|  | **Variation coefficient of the time** | 0.107 ± 0.047 (0.05-0.23) | 0.068 ± 0.031 (0.02-0.16) | <0.001 |
|  | **Variation coefficient of the velocity** | 0.087 ± 0.027 (0.05-0.17) | 0.058 ± 0.019 (0.03-0.12) | <0.001 |
|  | **Distortion index B-spline** | 1.06 ± 0.30 (0.56-1.84) | 0.58 ± 0.11 (0.40-0.99) | <0.001 |
|  | **Distortion index RCS** | 1.44 ± 0.40 (0.82-2.67) | 0.75 ± 0.16 (0.52-1.36) | <0.001 |
|  | **Distortion index POLY** | 1.14 ± 0.32 (0.61-1.88) | 0.62 ± 0.14 (0.45-1.22) | <0.001 |

Abbreviations: SCA = spinocerebellar ataxia; HC = healthy control; N.A. = not available; SARA = Scale for the Assessment and Rating of Ataxia; ICARS = International Cooperative Ataxia Rating Scale; RCS = restricted cubic spline; POLY = polynomial regression.

Data represent mean ± standard deviation (range). *Unpaired Student *t* test

**Supplementary Table 3.** Reliability and minimal detectable changes for the movement indices of the device

|  | **ICC (1,1) (95%CI)** | **SEM** | **MDC_95_** |
| --- | --- | --- | --- |
| **Trajectory length (mm)** | 0.967 (0.841 - 0.994) | 44 | 122 |
| **Time (sec)** | 0.963 (0.825 - 0.993) | 0.5 | 1.3 |
| **Velocity (mm/sec)** | 0.972 (0.866 - 0.995) | 15 | 42 |
| **Variation coefficient of the length** | 0.899 (0.573 – 0.982) | 0.005 | 0.013 |
| **Variation coefficient of the time** | 0.873 (0.483 – 0.976) | 0.012 | 0.033 |
| **Variation coefficient of the velocity** | 0.560 (-0.182 – 0.906) | 0.014 | 0.040 |
| **Distortion Index B-spline** | 0.984 (0.923 – 0.997) | 0.03 | 0.09 |
| **Distortion Index RCS** | 0.842 (0.390 – 0.970) | 0.17 | 0.47 |
| **Distortion Index POLY** | 0.987 (0.935 – 0.998) | 0.03 | 0.09 |

Abbreviations: ICC = Intraclass correlation coefficients; 95%CI = 95 per cent confidence intervals; SEM = standard error of measurement; MDC_95_ = minimal detectable change at the 95% confidence level; RCS = restricted cubic spline; POLY = polynomial regression.

**Supplementary Table 4.** Longitudinal analyses of functional measurements and indices in SCA patients with and without non-ataxia symptoms

|  | **SCA without non-ataxia symptoms (n = 7)** | | | | | |  | **SCA with non-ataxia symptoms (n = 23)** | | | | | |
| --- | --- | --- | --- | --- | --- | --- | --- | --- | --- | --- | --- | --- | --- |
|  | **Baseline**  **0W**  **(SD)** | **Follow-up 48W (SD)** | **Mean change (SD)** | ***p* Value*** | **SRM** | **aES** |  | **Baseline**  **0W**  **(SD)** | **Follow-up 48W (SD)** | **Mean change (SD)** | ***p* Value*** | **SRM** | **aES** |
| **SARA**  **total score** | 14.1  (3.7) | 14.2  (2.3) | 0.1  (2.9) | 0.899 | 0.05 | 0.04 |  | 14.4  (6.2) | 15.3  (6.1) | 1.0  (2.6) | 0.086 | 0.37 | 0.16 |
| **SARA upper limb score** | 4.4  (1.1) | 3.4  (0.4) | -1.0  (0.9) | 0.022 | -1.15 | -0.83 |  | 3.9  (1.8) | 4.1  (1.8) | 0.2  (1.1) | 0.382 | 0.19 | 0.11 |
| **ICARS**  **total score** | 31.6  (6.1) | 34.4  (7.7) | 2.9  (7.1) | 0.325 | 0.40 | 0.41 |  | 34.9  (15.0) | 37.2  (14.0) | 2.3  (5.4) | 0.059 | 0.42 | 0.15 |
| **ICARS upper limb score** | 7.7  (2.6) | 7.3  (2.1) | -0.4  (3.9) | 0.779 | -0.11 | -0.18 |  | 8.9  (3.8) | 9.1  (3.7) | 0.3  (2.7) | 0.652 | 0.10 | 0.07 |
| **9-hole peg test (sec)** | 41.8  (9.0) | 40.2  (6.2) | -1.6  (7.7) | 0.609 | -0.20 | -0.20 |  | 45.3  (22.5) | 45.8  (22.1) | 0.5  (13.0) | 0.850 | 0.04 | 0.02 |
| **Trajectory length (mm)** | 3768  (202) | 3848  (300) | 80  (243) | 0.419 | 0.33 | 0.30 |  | 3701  (302) | 3812  (422) | 112  (300) | 0.088 | 0.37 | 0.29 |
| **Time (sec)** | 11.2  (2.3) | 12.4  (1.5) | 1.2  (2.1) | 0.178 | 0.58 | 0.60 |  | 13.8  (5.5) | 13.5  (5.6) | -0.3  (2.1) | 0.494 | -0.15 | -0.05 |
| **Velocity (mm/sec)** | 354  (76) | 317  (41) | -36  (62) | 0.170 | -0.59 | -0.54 |  | 305  (96) | 325  (109) | 20  (34) | 0.011 | 0.58 | 0.18 |
| **Variation coefficient of the length** | 0.071  (0.015) | 0.074  (0.019) | 0.003  (0.016) | 0.622 | 0.20 | 0.19 |  | 0.064  (0.016) | 0.073  (0.024) | 0.009  (0.022) | 0.065 | 0.41 | 0.42 |
| **Variation coefficient of the time** | 0.096  (0.047) | 0.107  (0.049) | 0.012  (0.029) | 0.322 | 0.41 | 0.24 |  | 0.094  (0.024) | 0.097  (0.038) | 0.003  (0.026) | 0.564 | 0.12 | 0.09 |
| **Variation coefficient of the velocity** | 0.078  (0.023) | 0.093  (0.018) | 0.015  (0.026) | 0.175 | 0.58 | 0.72 |  | 0.080  (0.016) | 0.082  (0.020) | 0.002  (0.012) | 0.544 | 0.13 | 0.09 |
| **Distortion Index B-spline** | 0.93  (0.12) | 1.14  (0.13) | 0.21  (0.19) | 0.028 | 1.09 | 1.72 |  | 1.08  (0.33) | 1.24  (0.47) | 0.16  (0.33) | 0.028 | 0.49 | 0.37 |
| **Distortion Index RCS** | 1.53  (0.15) | 1.95  (0.50) | 0.42  (0.55) | 0.089 | 0.77 | 1.21 |  | 1.72  (0.48) | 1.90  (0.69) | 0.18  (0.50) | 0.101 | 0.36 | 0.28 |
| **Distortion Index POLY** | 1.03  (0.14) | 1.23  (0.16) | 0.19  (0.21) | 0.048 | 0.94 | 1.32 |  | 1.18  (0.35) | 1.32  (0.49) | 0.15  (0.33) | 0.045 | 0.44 | 0.32 |

Abbreviations: SCA = spinocerebellar ataxia; SARA = Scale for the Assessment and Rating of Ataxia; ICARS = International Cooperative Ataxia Rating Scale; RCS = restricted cubic spline; POLY = polynomial regression; SD = standard deviation, SRM = standardized response mean; aES = adjusted effect size.

Data represent mean (standard deviation). *Paired *t* test

**Supplementary Table 5.** Longitudinal analyses of functional measurements and indices in SCA patients at early and late stages

|  | **SCA with SARA total score ≤ 13.5 (n = 15)** | | | | | |  | **SCA with SARA total score > 13.5 (n = 15)** | | | | | |
| --- | --- | --- | --- | --- | --- | --- | --- | --- | --- | --- | --- | --- | --- |
|  | **Baseline**  **0W**  **(SD)** | **Follow-up 48W (SD)** | **Mean change (SD)** | ***p* Value*** | **SRM** | **aES** |  | **Baseline**  **0W**  **(SD)** | **Follow-up 48W (SD)** | **Mean change (SD)** | ***p* Value*** | **SRM** | **aES** |
| **SARA**  **total score** | 9.9  (3.2) | 11.7  (3.3) | 1.8  (1.9) | 0.003 | 0.93 | 0.56 |  | 18.7  (3.9) | 18.4  (5.1) | -0.2  (2.9) | 0.762 | -0.08 | -0.05 |
| **SARA upper limb score** | 3.0  (1.2) | 3.3  (1.3) | 0.2  (1.0) | 0.380 | 0.23 | 0.19 |  | 5.0  (1.5) | 4.6  (1.6) | -0.4  (1.2) | 0.212 | -0.34 | -0.26 |
| **ICARS**  **total score** | 24.4  (8.0) | 28.9  (10.9) | 4.5  (5.7) | 0.008 | 0.80 | 0.42 |  | 43.9  (10.2) | 44.1  (9.8) | 0.3  (5.1) | 0.843 | 0.05 | 0.03 |
| **ICARS upper limb score** | 6.6  (2.7) | 7.4  (3.3) | 0.8  (2.8) | 0.280 | 0.29 | 0.26 |  | 10.6  (3.1) | 10.0  (3.1) | -0.6  (3.1) | 0.468 | -0.19 | -0.19 |
| **9-hole peg test (sec)** | 34.7  (7.8) | 33.5  (7.1) | -1.2  (4.5) | 0.336 | -0.26 | -0.15 |  | 54.3  (23.8) | 55.6  (22.1) | 1.2  (16.4) | 0.777 | 0.07 | 0.05 |
| **Trajectory length (mm)** | 3670  (170) | 3800  (303) | 130  (241) | 0.055 | 0.54 | 0.48 |  | 3763  (359) | 3841  (475) | 78  (328) | 0.372 | 0.24 | 0.18 |
| **Time (sec)** | 10.1  (1.9) | 10.4  (2.4) | 0.2  (1.9) | 0.655 | 0.12 | 0.10 |  | 16.2  (5.5) | 16.1  (5.3) | -0.1  (2.5) | 0.850 | -0.05 | -0.02 |
| **Velocity (mm/sec)** | 380  (73) | 387  (81) | 7  (62) | 0.651 | 0.12 | 0.10 |  | 253  (63) | 259  (63) | 6  (28) | 0.451 | 0.20 | 0.09 |
| **Variation coefficient of the length** | 0.065  (0.016) | 0.077  (0.027) | 0.012  (0.022) | 0.057 | 0.54 | 0.49 |  | 0.066  (0.016) | 0.070  (0.018) | 0.003  (0.019) | 0.507 | 0.18 | 0.20 |
| **Variation coefficient of the time** | 0.096  (0.029) | 0.105  (0.041) | 0.010  (0.034) | 0.285 | 0.29 | 0.26 |  | 0.093  (0.032) | 0.094  (0.040) | 0.001  (0.017) | 0.870 | 0.04 | 0.02 |
| **Variation coefficient of the velocity** | 0.071  (0.014) | 0.080  (0.018) | 0.009  (0.020) | 0.111 | 0.44 | 0.54 |  | 0.089  (0.017) | 0.089  (0.021) | 0.001  (0.013) | 0.822 | 0.06 | 0.04 |
| **Distortion Index B-spline** | 0.93  (0.18) | 1.12  (0.40) | 0.19  (0.35) | 0.048 | 0.56 | 0.56 |  | 1.16  (0.35) | 1.31  (0.42) | 0.15  (0.26) | 0.038 | 0.59 | 0.38 |
| **Distortion Index RCS** | 1.56  (0.28) | 1.86  (0.60) | 0.30  (0.58) | 0.066 | 0.52 | 0.60 |  | 1.79  (0.53) | 1.96  (0.69) | 0.17  (0.45) | 0.158 | 0.38 | 0.27 |
| **Distortion Index POLY** | 1.04  (0.20) | 1.20  (0.42) | 0.16  (0.35) | 0.095 | 0.46 | 0.43 |  | 1.25  (0.39) | 1.40  (0.45) | 0.15  (0.26) | 0.039 | 0.59 | 0.36 |

Abbreviations: SCA = spinocerebellar ataxia; SARA = Scale for the Assessment and Rating of Ataxia; ICARS = International Cooperative Ataxia Rating Scale; RCS = restricted cubic spline; POLY = polynomial regression; SD = standard deviation, SRM = standardized response mean; aES = adjusted effect size.

Data represent mean (standard deviation). *Paired *t* test

**Supplementary Table 6.** Longitudinal analyses of functional measurements and indices in SCA3 patients (n = 7)

|  | **Baseline**  **0W (SD)** | **Follow-up 48W (SD)** | **Mean longitudinal change (SD)** | ***p* Value*** | **SRM** | **aES** |
| --- | --- | --- | --- | --- | --- | --- |
| **SARA total score** | 12.7 (8.1) | 12.1 (7.4) | -0.6 (3.5) | 0.679 | -0.16 | -0.07 |
| **SARA upper limb score** | 2.9 (1.8) | 3.1 (2.1) | 0.2 (0.9) | 0.534 | 0.25 | 0.10 |
| **ICARS total score** | 32.0 (19.9) | 31.4 (18.9) | -0.6 (5.3) | 0.783 | -0.11 | -0.03 |
| **ICARS upper limb score** | 5.9 (4.7) | 7.4 (4.9) | 1.6 (3.4) | 0.268 | 0.46 | 0.33 |
| **9-hole peg test (sec)** | 37.0 (16.0) | 39.9 (25.9) | 2.9 (10.4) | 0.494 | 0.27 | 0.04 |
| **Trajectory length (mm)** | 3762 (150) | 3880 (531) | 118 (441) | 0.507 | 0.27 | 0.21 |
| **Time (sec)** | 11.9 (5.0) | 11.6 (7.0) | -0.4 (2.5) | 0.711 | -0.15 | -0.04 |
| **Velocity (mm/sec)** | 354 (107) | 395 (126) | 41 (48) | 0.066 | 0.85 | 0.32 |
| **Variation coefficient of the length** | 0.055 (0.015) | 0.067 (0.019) | 0.012 (0.025) | 0.250 | 0.48 | 0.68 |
| **Variation coefficient of the time** | 0.086 (0.025) | 0.091 (0.034) | 0.005 (0.028) | 0.655 | 0.18 | 0.16 |
| **Variation coefficient of the velocity** | 0.073 (0.014) | 0.072 (0.019) | 0.000 (0.012) | 0.943 | -0.03 | -0.02 |
| **Distortion Index B-spline** | 0.95 (0.28) | 1.05 (0.55) | 0.11 (0.32) | 0.412 | 0.33 | 0.14 |
| **Distortion Index RCS** | 1.59 (0.38) | 1.74 (0.89) | 0.15 (0.58) | 0.517 | 0.26 | 0.13 |
| **Distortion Index POLY** | 1.04 (0.30) | 1.14 (0.58) | 0.10 (0.32) | 0.448 | 0.31 | 0.11 |

Abbreviations: SCA = spinocerebellar ataxia; SARA = Scale for the Assessment and Rating of Ataxia; ICARS = International Cooperative Ataxia Rating Scale; RCS = restricted cubic spline; POLY = polynomial regression; SD = standard deviation, SRM = standardized response mean; aES = adjusted effect size.

Data represent mean (standard deviation). *Paired *t* test

**Supplementary Table 7.** Longitudinal analyses of functional measurements and indices in SCA6 patients (n = 6)

|  | **Baseline**  **0W (SD)** | **Follow-up 48W (SD)** | **Mean longitudinal change (SD)** | ***p* Value*** | **SRM** | **aES** |
| --- | --- | --- | --- | --- | --- | --- |
| **SARA total score** | 14.3 (4.4) | 13.9 (3.1) | -0.3 (2.3) | 0.737 | -0.15 | -0.08 |
| **SARA upper limb score** | 4.1 (0.9) | 3.9 (1.1) | -0.2 (1.1) | 0.732 | -0.15 | -0.17 |
| **ICARS total score** | 31.5 (11.9) | 34.0 (10.0) | 2.5 (5.1) | 0.286 | 0.49 | 0.21 |
| **ICARS upper limb score** | 7.7 (3.4) | 7.8 (1.3) | 0.2 (2.4) | 0.872 | 0.07 | 0.04 |
| **9-hole peg test (sec)** | 45.5 (19.9) | 49.8 (26.2) | 4.3 (6.5) | 0.168 | 0.66 | 0.04 |
| **Trajectory length (mm)** | 3591 (256) | 3691 (441) | 101 (289) | 0.432 | 0.35 | 0.23 |
| **Time (sec)** | 12.6 (6.3) | 12.7 (4.1) | 0.1 (3.4) | 0.960 | 0.02 | 0.01 |
| **Velocity (mm/sec)** | 334 (128) | 318 (102) | -15 (79) | 0.652 | -0.20 | -0.13 |
| **Variation coefficient of the length** | 0.060 (0.016) | 0.059 (0.013) | -0.001 (0.015) | 0.877 | -0.07 | -0.07 |
| **Variation coefficient of the time** | 0.084 (0.028) | 0.102 (0.026) | 0.018 (0.035) | 0.270 | 0.51 | 0.65 |
| **Variation coefficient of the velocity** | 0.073 (0.016) | 0.086 (0.021) | 0.013 (0.027) | 0.301 | 0.47 | 0.70 |
| **Distortion Index B-spline** | 0.96 (0.28) | 1.16 (0.28) | 0.20 (0.24) | 0.104 | 0.81 | 0.70 |
| **Distortion Index RCS** | 1.51 (0.35) | 1.82 (0.48) | 0.30 (0.38) | 0.110 | 0.79 | 0.69 |
| **Distortion Index POLY** | 1.04 (0.31) | 1.23 (0.30) | 0.20 (0.26) | 0.126 | 0.75 | 0.65 |

Abbreviations: SCA = spinocerebellar ataxia; SARA = Scale for the Assessment and Rating of Ataxia; ICARS = International Cooperative Ataxia Rating Scale; RCS = restricted cubic spline; POLY = polynomial regression; SD = standard deviation, SRM = standardized response mean; aES = adjusted effect size.

Data represent mean (standard deviation). *Paired *t* test

**Supplementary Table 8.** Longitudinal analyses of functional measurements and indices in SCA31 patients (n = 8)

|  | **Baseline**  **0W (SD)** | **Follow-up 48W (SD)** | **Mean longitudinal change (SD)** | ***p* Value*** | **SRM** | **aES** |
| --- | --- | --- | --- | --- | --- | --- |
| **SARA total score** | 14.0 (3.8) | 15.4 (2.9) | 1.4 (1.7) | 0.050 | 0.84 | 0.38 |
| **SARA upper limb score** | 4.4 (1.0) | 3.8 (0.8) | -0.6 (1.1) | 0.185 | -0.52 | -0.64 |
| **ICARS total score** | 34.4 (10.5) | 39.3 (9.0) | 4.9 (6.0) | 0.055 | 0.81 | 0.49 |
| **ICARS upper limb score** | 10.5 (1.6) | 9.5 (3.1) | -1.0 (2.7) | 0.334 | -0.37 | -0.38 |
| **9-hole peg test (sec)** | 40.3 (6.9) | 40.1 (6.9) | -0.2 (5.9) | 0.918 | -0.04 | -0.03 |
| **Trajectory length (mm)** | 3607 (215) | 3683 (215) | 76 (142) | 0.173 | 0.54 | 0.35 |
| **Time (sec)** | 12.2 (2.4) | 12.7 (2.5) | 0.5 (0.8) | 0.116 | 0.63 | 0.21 |
| **Velocity (mm/sec)** | 311 (62) | 304 (59) | -7 (14) | 0.197 | -0.50 | -0.12 |
| **Variation coefficient of the length** | 0.071 (0.016) | 0.079 (0.021) | 0.009 (0.014) | 0.137 | 0.59 | 0.43 |
| **Variation coefficient of the time** | 0.104 (0.038) | 0.105 (0.046) | 0.001 (0.012) | 0.855 | 0.07 | 0.02 |
| **Variation coefficient of the velocity** | 0.085 (0.019) | 0.086 (0.019) | 0.002 (0.018) | 0.797 | 0.09 | 0.09 |
| **Distortion Index B-spline** | 1.01 (0.12) | 1.17 (0.23) | 0.16 (0.15) | 0.017 | 1.10 | 0.66 |
| **Distortion Index RCS** | 1.62 (0.21) | 1.82 (0.34) | 0.21 (0.23) | 0.040 | 0.89 | 0.64 |
| **Distortion Index POLY** | 1.13 (0.11) | 1.26 (0.23) | 0.13 (0.19) | 0.092 | 0.69 | 0.62 |

Abbreviations: SCA = spinocerebellar ataxia; SARA = Scale for the Assessment and Rating of Ataxia; ICARS = International Cooperative Ataxia Rating Scale; RCS = restricted cubic spline; POLY = polynomial regression; SD = standard deviation, SRM = standardized response mean; aES = adjusted effect size.

Data represent mean (standard deviation). *Paired *t* test
